# Single center experience and early outcomes of Impella 5.5

**DOI:** 10.3389/fcvm.2023.1018203

**Published:** 2023-02-28

**Authors:** Masaki Funamoto, Chandra Kunavarapu, Michael D. Kwan, Yuichi Matsuzaki, Mahek Shah, Masahiro Ono

**Affiliations:** ^1^Department of Cardiothoracic Surgery, Methodist Hospital, San Antonio, TX, United States; ^2^Advanced Heart Failure and Transplant Cardiology, Methodist Hospital, San Antonio, TX, United States

**Keywords:** cardiogenic shock, Impella, temporary mechanical circulatory support, bridge to recovery, bridge to transplant, bridge to LVAD, high risk cardiac surgery

## Abstract

**Background:**

Acute decompensated heart failure (HF) and cardiogenic shock (CS) frequently are refractory to conservative treatment and require mechanical circulatory support (MCS). We report our early clinical experience and evaluate patient outcomes with the newer generation surgical Impella 5.5.

**Methods:**

Seventy patients that underwent Impella 5.5 implantation between October 2019 and December 2021 at a single center were enrolled in this study. Pre-operative characteristics, peri-operative clinical course information, and post-operative outcomes were retrospectively collected.

**Results:**

Fifty-seven (81%) patients survived to discharge, and 51 (76%) patients survived at the time of the first 30 days post-discharge visit. Thirty-one patients (44%) received Impella support for a bridge to advanced surgical heart failure therapy (transplant or durable left ventricular assist device [LVAD]), 27 (39%) cases were used for a bridge to recovery/decision and 12 (17.1%) cases was used for planned perioperative support for high-risk cardiac surgery procedure.

**Conclusion:**

Our results suggest that Impella 5.5 provides favorable survival in the management of HF and CS, particularly used for a bridge to heart transplant or LVAD. Early extubation and mobilization with high flow circulatory support allowed effective tailoring of MCS approaches from peri-operative support for high-risk cardiac surgery, bridge to recovery, and to advanced surgical heart failure therapy.

## Introduction

Cardiogenic shock (CS) is associated with in-hospital mortality rates ranging from 27 to 51%, and management remains challenging despite advances in therapies (1–4). Cardiogenic shock is caused by severe impairment of the myocardium that results in diminished cardiac output, end-organ hypoperfusion, and hypoxia. While inotropic agents are widely used, mortality is higher with an increased number of prescribed vasopressors. Catecholamine therapy is associated with significant limitations including arrhythmias, increased myocardial oxygen consumption, and inadequate circulatory support (5, 6). Temporary mechanical circulatory support (MCS) is a key component of early patient management for CS with pronounced benefits, including substantial cardiovascular support without increased risk of myocardial ischemia and possible decreased myocardial oxygen demand, which may increase the likelihood of eventual recovery. Registry data indicate that early MCS device use is associated with improved rates of survival rather than deferred use in acute myocardial infarction CS (7, 8). There are various options for acute percutaneous MCS: the intra-aortic balloon pump (IABP), axial flow pumps/catheter-based left ventricular assist device (cVAD; Impella 2.5, Impella CP), left atrial-to-femoral arterial ventricular assist devices (Tandem Heart), and venous–arterial extracorporeal membrane oxygenation (ECMO) (9–12). These devices are designed for rapid deployment, short term support, requiring bed-rest, which often affects mobility and recovery. IABP can be placed *via* the axillary artery, but often is insufficient to support patients with profound CS, and has not demonstrated an early mortality benefit for patients with CS (1).

Impella 5.5 is a microaxial, surgically implanted heart pump that unloads the left ventricle, reduces ventricular work, and provides the circulatory support necessary to allow recovery and early assessment of residual myocardial function. It is designed for long-duration support and enables ambulation to optimize recovery while using real-time SmartAssist intelligence (13).

In October 2018, a new heart transplant allocation system was implemented with a 6-tiered classification system in the United States. Non-dischargeable mechanical support devices such as IABP, cVADs, and ECMO classified patients as Status 1 or 2, and are prioritized under the new system (13), with ambulatory Impella 5.5 in the spotlight as a favorable bridging strategy for heart transplant. Here, we report early outcomes in patients implanted with the Impella 5.5 at a single-center and current clinical use of axillary Impella in a mid-America tertiary high-volume MCS medical center.

## Materials and methods

### Study design

Patients that underwent the Impella 5.5 implantation at Methodist Hospital San Antonio, TX, between October 2019 and December 2021 were included in the study. The Surgical Unloading Renal Protections and Sustainable Support Study (SURPASS) registry (NCT05100836) is a database for all surgically implanted Impella procedures that is prospectively maintained by the manufacturer and retrospective collection of data. Clinical and outcome data were obtained from this SURPASS registry and retrospective review of electronic medical records. Patients were followed after hospital discharge for recovery, death, or transition to durable left ventricular assist device (LVAD) or heart transplantation. This study was approved by the Hospital Corporation of American (HCA) Healthcare Institutional Review Board. The investigation conforms with the principles outlined in the Declaration of Helsinki.

### Device design, surgical technique, and mechanical circulatory support strategy

The Impella 5.5^®^ with SmartAssist^®^ heart pump is a temporary LVAD intended for longer use, with FDA approval for up to 14 days and CE mark approval for up to 30 days. The device can be inserted either *via* the axillary artery or by direct aortic access with a minimum vessel diameter ≥ 7 mm. The inlet position on the Impella 5.5 differs from earlier models and must be positioned 5 cm below the aortic valve annulus. The device is equipped with optical sensor technology, to be used with echocardiography to facilitate proper device positioning across the aortic valve.

All patients underwent Impella implantation under general anesthesia. The intraoperative echocardiogram and pulmonary artery line was used to monitor cardiac function. The Impella 5.5 was inserted at the axillary artery, which is suitable for longer support without concern of mediastinal infection. If pre-op CT is available, we recommend checking the diameter of the axillary artery to ensure it is 6 mm or more, as well as to examine for calcification, stenotic, or tortuous vessels and arch, and the length of ascending aorta with 7 cm or more for access site evaluation. In addition, use of preoperative transthoracic echocardiogram (TTE) allows visualization of LV cavity size, angle of the LV-aortic root and assessment of aortic valve leaflet calcification/atherosclerosis. Although an infrequent occurrence, aortic valve leaflet calcification/atherosclerosis can cause Impella related aortic insufficiency, thus providing an opportunity to predict the potential device related risk.

For axillary approach, the pectoralis major was divided along with muscle fiber, and the pectoralis minor was retracted laterally without severing muscles to prevent potential bleeding from the muscle surface. The thoracoacromial artery was used as an important anatomical landmark to locate the axillary artery with minimal dissection. After exposing the axillary artery, heparin is administered to obtain an activated clotting time >250s. Our standard surgical approach for Impella 5.5 was established with a 10 mm prosthetic vascular graft, anastomosed in an end-to-side fashion to the right axillary artery. The access site was determined based on a CT scan. We prefer to use right axillary approach for axillary Impella since catheters make a natural curve and go straight down to the ascending aorta and LV in most of the cases. For axillary IABP, the left axillary approach is preferred to avoid excess stress on the intima of ascending aorta/arch vessels, especially for longer term use. However, if the right axillary approach is not feasible or there is any visible tortuous arch vessels *via* CT scan, the left axillary artery approach should be considered, with the ascending aorta or the innominate artery as alternatives for Impella access. Insertion was guided under fluoroscopy and positioning was adjusted using intraoperative transesophageal echocardiography.

**Figure 1:**
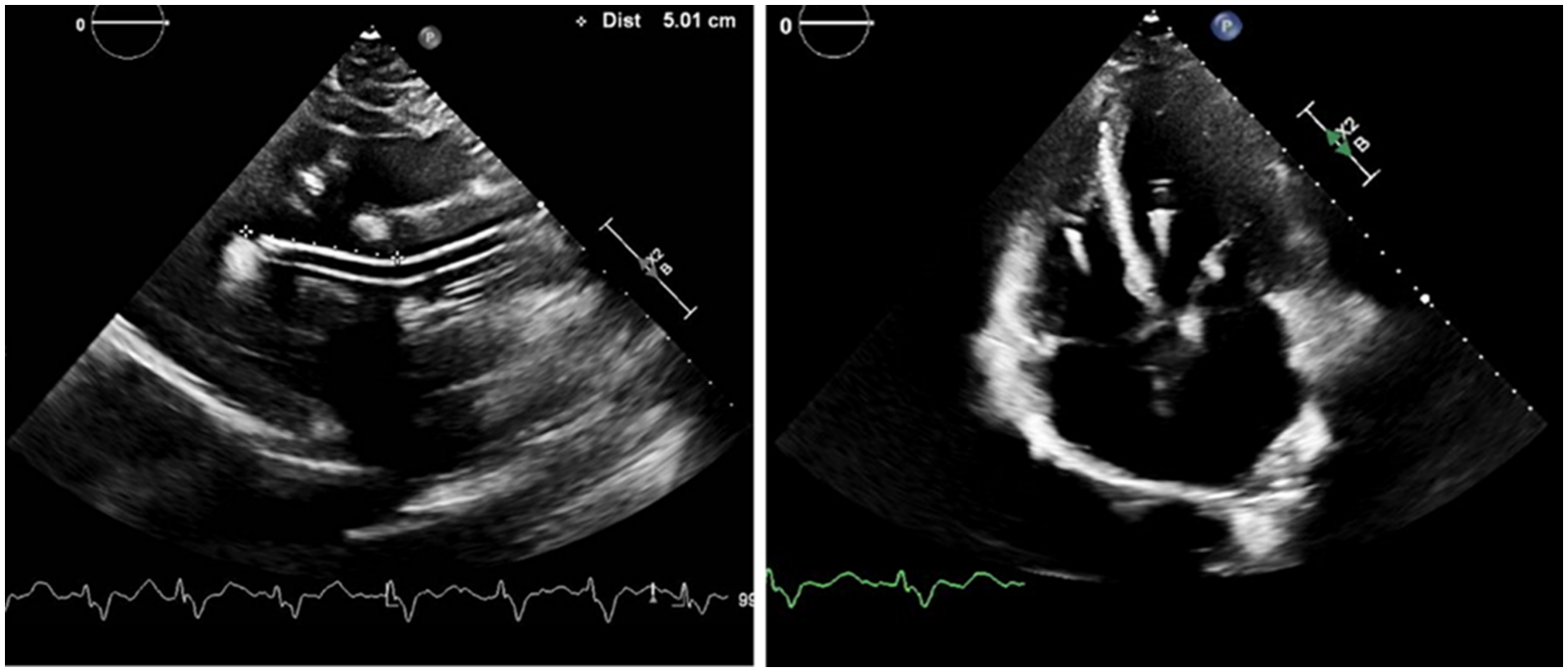
Positioning of the Impella 5.5: The bending portion of Impella pump head will be on the level of aortic valve with the tip pointing away from the posterior wall in the parasternal long axis view, and away from septum in the four chamber view. Impella position-related aortic insufficiency could be observed for patients with atherosclerotic aortic valve in the apical view.

Once inserted, at least two views of a TTE, such as parasternal long axis and apical four chamber view, are obtained to confirm the Impella position. Impella 5.5 does not have a pigtail and thus, it is easy to change the position, but as a result there is also higher risk of the device moving from its ideal positioning. Moreover, the device could attach to or push the septum if rotated completely away from the mitral valve and could lead to suction events. Therefore, the Impella pump needs to be away from the LV wall in multiple views (Figure 1).

For patients with cardiogenic shock, MCS utilization is considered when two or more moderate doses of inotropes or vasopressors are needed. We prefer to first use dobutamine, add epinephrine as a second agent, and then use vasopressin/norepinephrine bitartrate as needed to maintain cardiac output and perfusion pressure for cardiogenic shock. The threshold of MCS is lowered if the patient has an arrhythmia issue. After MCS initiation, vasopressors are decreased as much as possible, and inotropes are titrated according to the hemodynamic/perfusion status, but a moderate dose of dobutamine (3–5 mcg/kg/min) is usually maintained for RV support.

The combination of Impella 5.5 and veno-arterial extracorporeal membrane oxygenation (VA-ECMO) is established as a stepped strategy. As such, we do not suggest Impella 5.5 as a rescue device. For profound CS requiring VA-ECMO, Impella CP or IABP are used for LV unloading or afterload reduction at the first line additional support, to be transitioned to Impella 5.5.

### Data collection and statistical analysis

Data were collated and tabulated using Microsoft Excel (Microsoft Corporation, Redmond, WA, United States). Normally distributed descriptive statistics are presented as mean (quartile 1 (Q1), quartile 3 (Q3)) for continuous variables and *n* (percent) for categorical variables. Tabular denominators reflect the number with available data for a given data point. Survival analyzes were performed using Kaplan–Meier analyzes. Independent variables with >10% unrecorded or missing values were excluded, with the majority of factors in the tables having no missing data. Statistical analyzes were performed using GraphPad Prism v9 (GraphPad Software Inc., San Diego, CA, United States).

## Results

### Patient population and procedural characteristics

Patients in our study cohort were 90% male and 55 years of age (Q1–Q3, 48–65). Baseline left ventricular ejection fraction (LVEF) was 20% (Q1–Q3, 15–22) and 48 (69%) patients had at least mild right ventricular dysfunction by TTE. The etiologies requiring MCS were acute myocardial infarction (17%), acute decompensated heart failure (HF, 61%), post-cardiotomy CS (4%), and planned support for high-risk cardiac surgery (17%). The indications of Impella implantation were bridge to decision in 7 (10%), bridge to recovery in 20 (29%), bridge to durable LVAD in 6 (9%) cases, bridge to heart transplant in 25 (36%) cases, and perioperative support for high-risk cardiac surgery in 12 (17%) cases. Fifty patients (71%) had other types of MCS prior to Impella 5.5 implantation. Thirty (44%) patients were upgraded to Impella 5.5 from intra-aortic balloon pump (IABP), 16 (25%) from Impella CP percutaneous LVAD, and 14 (20%) patients were transitioned to 5.5 as a de-escalation from VA-ECMO. Baseline and procedural characteristics are presented in Table 1.

**Table 1:** Baseline characteristics and periprocedural data.

Baseline clinical characteristics	All (*n* = 70)	Bridge to recovery/decision (*n* = 27)	Bridge to heart transplant/LVAD (*n* = 31)	Planned perioperative support (*n* = 12)
Age, years	55.4 (48.3, 65.0)	59.0 (57.0, 65.5)	50.4 (37.0, 63.5)	60.2 (56.5, 65.3)
Male, *n* (%)	63 (90)	23 (85)	29 (94)	11 (92)
BMI, kg/m^2^	29.0 (25.5, 32.2)	30.5 (25.9, 34.5)	28.5 (25.3, 30.9)	26.7 (25.2, 28.7)
Cardiogenic Shock, *n* (%)	58 (83)	26 (96)	27 (87)	5 (42)
Post-CPR, *n* (%)	13 (19)	11 (41)	2 (7)	0 (0)
ICM, *n* (%)	36 (51)	19 (70)	9 (29)	8 (67)
Baseline LVEF, %	20.1 (5.4, 6.8)	24.0 (17.9, 30.7)	16.2 (14.0, 20.0)	21.1 (16.3, 26.5)
Baseline LVEDD, mm	6.1 (5.4, 6.8)	5.8 (5.0, 6.5)	6.5 (6.0, 6.9)	6.0 (5.3, 6.5)
Severe mitral regurgitation, *n* (%)	16 (23)	7 (26)	7 (23)	2 (17)
RV dysfunction^*^, *n* (%)	48 (69)	19 (70)	26 (84)	3 (25)
SCAI stages at admission				
A: “At risk”	2 (3)	0 (0)	0 (0)	2 (17)
B: “Beginning” cardiogenic shock	2 (3)	0 (0)	0 (0)	2 (17)
C: “Classic” cardiogenic shock	39 (56)	8 (30)	26 (84)	5 (42)
D: “Deteriorating” cardiogenic shock	15 (21)	8 (30)	4 (13)	3 (25)
E: “Extremis”	12 (17)	11 (16)	1 (3)	0 (0)
Pre-op Creatinine	1.9 (1.1, 2.2)	2.1 (1.2, 2.6)	1.9 (1.3, 2.2)	1.2 (1.0, 1.2)
Pre-op ALT	281.0 (28.3, 134.5)	588.0 (37.5, 280.0)	100.0 (21.5, 105.0)	57.7 (28.8, 67.3)
MCS prior to Impella 5.5, *n* (%)	50 (71)	24 (89)	22 (71)	5 (46)
Impella 5.5 added to VA ECMO, *n* (%)	14 (20)	8 (30)	6 (19)	0 (0)
Upgrade from IABP, *n* (%)	30 (44)	10 (37)	19 (61)	2 (17)
Upgrade from Impella 2.5/CP, *n* (%)	16 (23)	13 (48)	1 (3)	2 (17)
Impella insertion site				
Right Axillary artery, *n* (%)	66 (94)	28 (100)	31 (100)	8 (67)
Ascending aorta, *n* (%)	3 (4)	0 (0)	0 (0)	3 (25)
Innominate artery, *n* (%)	1 (1)	0 (0)	0 (0)	1 (8)

Data is presented as *n* (%) or mean (quartile 1, quartile 3). ALT, alanine transaminase; BMI, body mass index; CPR, cardiopulmonary resuscitation; IABP, intra-aortic balloon pump; ICM, ischemic cardiomyopathy; LVAD, left ventricular assist device; LVEF, left ventricular ejection fraction; MCS, mechanical circulatory support; SD, standard deviation; VA ECMO, venoarterial extracorporeal membrane oxygenation ^*^RV dysfunction was defined if it meets at least one of the following values: (I) tricuspid annular place systolic excursion (TAPSE) < 17 mm; (II) fractional area change (FAC) < 35%; (III) and/or tricuspid annular systolic velocity (s’) < 9.5 cm/s, according to the recommendation guidelines from ASE/EAC (14).

### Outcomes

The majority of patients had Impella 5.5 inserted *via* the right axillary artery (96%). The mean duration of Impella support was 10 days (Q1–Q3, 6–12 days). For the cohort, overall survival to discharge was 57 patients (81%). Five patients (7%) were bridged to durable LVAD, 23 patients (33%) received heart transplantation, and 29 patients (41%) achieved cardiac recovery (Table 2). Of the patients in this study, survival to discharge occurred in 13/18 (72%) for bridge to recovery, 4/9 (44%) for bridge to decision, 23/25 (92%) for bridge to heart transplant, 5/6 (83%) for bridge to durable surgical LVAD, and 12/12 (100%) for bridge to recovery from high-risk cardiac surgery. In the group of bridge to recovery/decision and bridge to heart transplant/LVAD, the majority of patients had concomitant RV systolic dysfunction by TTE (70 and 84%, respectively).

**Table 2:** Clinical outcomes on Impella Support.

Clinical outcome on Impella support	All (*n* = 70)	Bridge to recovery/decision (*n* = 27)	Bridge to heart transplant/LVAD (*n* = 31)	Planned perioperative support (*n* = 12)
Duration of Impella 5.5 support, days	10.0 (6.0, 12.0)	7.4 (5.0, 10.5)	13.6 (8.0, 17.0)	6.4 (5.8, 7.3)
Length of hospital stay, days	27.5 (17.0, 34.8)	17.7 (15.0, 21.0)	38.6 (24.5, 42.0)	20.9 (14.8, 25.3)
Survival to discharge, *n*/*N* (%)	57/70 (81)	17/27 (63)	29/31 (94)	12/12 (100)
Post discharge 30 days survival^*^, *n*/*N* (%)	52/68 (77)	13/26 (50)	29/31 (94)	11/11 (100)

Data is presented as *n* /N (%) or mean (quartile 1, quartile 3). ^*^After the index admission. LVAD, left ventricular assisted device.

Notably, in the bridge to transplant group, 10 patients receiving Impella were bridged from IABP due to support failure. In addition, 44% of patients who underwent Impella 5.5 placement were transitioned from IABP, which includes upgrading for IABP support failure and de-escalation from IABP/VA-ECMO.

With respect to Impella-supported cardiac surgery, seven patients underwent isolated coronary artery bypass grafting, and five cases were valve or combined valve/coronary/aortic procedures. In a total of 10 STS cases, the mean STS scores of mortalities were 5.2% (Q1–Q3, 1.6–7.5%). All patients survived to discharge from the index admission (Table 3).

**Table 3:** Outcomes for Impella-supported cardiac surgery.

Case	Gender	BMI	SCAI at Admission	RV Failure	Pre-Op Cr	Pre-op LVEF (%)	Pre-op LVED (cm)	Previous MCS Devices	Access Approach	STS Score Mortality (%)	STS Score Major Morbidity (%)	Total ischemic time (min)	Total CPB time (min)	Cardiac Procedure	Duration of support (days)	Post-discharge 30-day survival
1	Male	29.4	B	NO	1.01	16.7	6.5	No	Rt. Axillary	3.65	17.9	68	104	On-pump CABG	8	Yes
2	Male	30.3	A	YES	0.79	32.3	5.8	No	Rt. Axillary	1.30	14.8	93	132	MVR	4	Yes
3	Male	27.0	B	YES	0.86	15.0	4.5	No	Rt. Axillary	2.47	16.3	63	91	On-pump CABG	6	Yes
4	Male	24.5	C	YES	1.00	13.8	6.2	No	Direct Aorta	7.03	30.1	69	122	On-pump CABG	8	Lost to FU
5	Male	28.5	C	NO	1.08	28.0	6.6	No	Direct Aorta	0.61	6.8	84	126	On-pump CABG	7	Yes
6	Male	27.1	C	NO	1.17	23.0	4.7	No	Innominate	N/A	N/A	142	323	AVR; ascending aortic replacement	8	Yes
7	Male	25.7	D	NO	2.09	19.0	6.0	Impella CP	Rt. Axillary	14.24	56.6	49	68	On-pump CABG	7	Yes
8	Male	20.0	C	NO	0.96	19.0	7.8	No	Direct Aorta	N/A	N/A	123	226	MV repair; AVR	7	Yes
9	Male	25.4	A	NO	0.90	29.0	7.0	No	Rt. Axillary	0.97	12.3	67	95	MVR	4	Yes
10	Male	32.2	C	NO	0.98	18.0	5.3	IABP	Rt. Axillary	7.60	74.0	115	147	On-pump CABG; AVR	6	Yes
11	Male	27.1	D	NO	1.87	13.6	6.0	Impella CP	Rt. Axillary	8.70	63.0	79	104	On-pump CABG	7	Yes
12	Female	22.8	D	NO	1.35	26.0	5.1	IABP; Impella 2.5	Rt. Axillary	5.20	42.0	96	121	On-pump CABG	5	Yes

AVR, aortic valve replacement; BMI, body mass index; CABG, coronary artery bypass graft; cm, centimeter; CPB, cardiopulmonary bypass; FU, follow-up; IABP, intra-aortic balloon pump; LVEF, left ventricle ejection fraction; LVED, left ventricular end diastolic; MCS, mechanical circulatory support; min, minute; MVR, mitral valve replacement; N/A, not applicable; Pre-op, pre-operation; RV, right ventricle; SCAI, Society for Cardiovascular Angiography and Interventions; STS, Society of Thoracic Surgeons.

**Figure 2:**
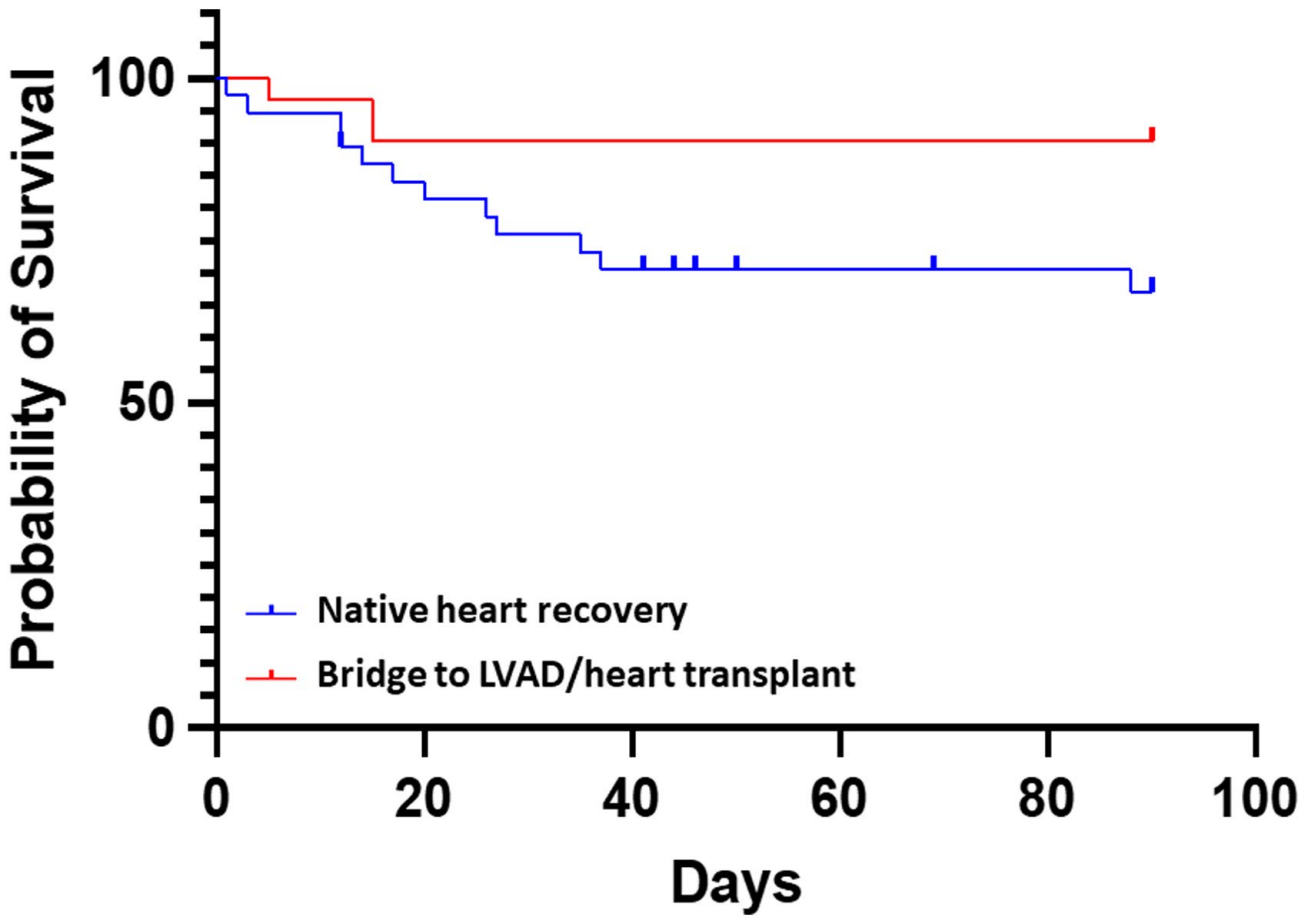
Comparative survival for patients receiving Impella 5.5. The probability of survival was greater in patients with a bridge to LVAD/heart transplant vs. bridge to native heart recovery (non LVAD/heart transplant) HR 3.71, 95% CI 1.35–10.21; *p* = 0.029.

As shown in Figure 2, Kaplan–Meier analysis demonstrated significant difference in 90 days survival between the groups of bridge to LVAD/heart transplant and non LVAD/heart transplant (native heart recovery, HR 3.71, 95% CI 1.35–10.21; *p* = 0.029). Thirty-two patients (46%) experienced at least one complication while on Impella 5.5 support. Four patients had thrombocytopenia, 6 developed axillary hematomas requiring exploration, 1 had mediastinal bleeding requiring chest washout, 6 had acute kidney injury or required renal replacement therapy, and 2 patients had cerebrovascular accidents. There were 7 patients with transient high plasma free hemoglobin (pf-Hb) level (>20 mg/dL), all of which were resolved with positional or anticoagulation adjustment, and only 1 patient had device dislodgement requiring revision of Impella 5.5 placement. There was one surgical site infection requiring vascular construction. Aortic valve injury, distal limb ischemia, or other vascular complications did not occur (Table 4).

**Table 4:** Adverse events related to Impella 5.5.

Complication	*N* (%)
Stroke	2 (2.9)
Acute kidney injury	3 (4.3)
Renal replacement therapy	3 (4.3)
Acute hepatic dysfunction	2 (2.9)
Respiratory failure/dysfunction^*^	4 (5.7)
Thrombocytopenia^**^	4 (5.7)
Anemia	10 (14.3)
Bleeding requiring surgery	7 (10.0)
Surgical site infection	1 (1.4)
Valve injury	0 (0)
Cardiac perforation	0 (0)
Ventricular arrhythmia	6 (8.6)
Device dislodgement	1 (1.4)
Hemolysis (Pf-Hb > 20 mg/dL)	7 (10.0)

Pf-Hb: plasma free hemoglobin. ^*^Respiratory failure/dysfunction is defined as the impairment of respiratory function requiring reintubation, tracheostomy or the inability to discontinue ventilatory support 48 h after Impella device explant. This excludes intubation for reoperation or temporary intubation for diagnostic or therapeutic procedures. ^**^Thrombocytopenia is defined as having a platelet count measurement of less than 50,000/mm^3^ taken more than 48 h after the Impella implant.

## Discussion

While studies using Impella devices for CS show reasonable survival for AMI-CS (15), several smaller randomized clinical trials have failed to demonstrate improved outcomes with Impella 2.5 or Impella CP over IABP (9, 15–17). However, the first 200 cases in the US using Impella 5.5 had a rate of overall survival to explant of 74% in patients with CS (18). The current single center study further supports the potential benefit of Impella 5.5 for CS management with 81% of the overall survival to discharge after Impella 5.5 implantation. Of these patients, 74% had other types of MCS prior to Impella, 44% received Impella support for a bridge to advanced surgical heart failure therapy, 39% of cases were for a bridge to recovery/decision, and 17% cases were perioperative support for high-risk cardiac surgery. Moreover, all patients receiving a heart transplant from Impella 5.5 bridge survived more than 90 days after discharge. Thus, future studies are warranted with the newer Impella devices (5.0/5.5), which have higher flow rates and may provide more sufficient cardiac output to maintain systemic organ perfusion in patients with severe CS or heart failure requiring full hemodynamic support.

Outcomes for CS with medical management without any mechanical circulatory support is poor, particularly for patients with prominent CS, stage D or E according to the Society of Cardiovascular Angiography and Interventions (SCAI) CS classifications (19–21). As such, temporary MCS devices are often used as a bridge until the patient recovers or condition deteriorates needing a long-term assist device or heart transplantation. IABP was often implemented as a first-line temporary MCS for refractory CS and acute decompensated heart failure, due to its quick deployment and less invasive features compared with other MCS devices. As such, IABP did not show survival benefit for cardiogenic shock due to acute myocardial infarction (AMI-CS) (22). However, several observational studies have indicated that the IABPs can improve outcome in cardiogenic shock due to acute decompensated heart failure (HF-CS). Compared to AMI-CS, it has a different underlying pathophysiology and, accordingly, different responses to pharmacological treatments and mechanical support (23, 24). IABP combines a more substantial effect on left ventricular afterload with a modest increase (0.5–1.0 L/min; 25, 26) in cardiac output and would therefore be most suitable in clinical scenarios characterized by a disproportionate increase in afterload without profound hemodynamic compromise. For a bridge to transplant/LVAD, groin IABP is switched to axillary position once stabilized with groin IABP so that patients can ambulate while waiting for transplant/LVAD. However, some patients fail to stabilize with IABP due to insufficient support or multiple dislodgement in axillary IABP. Our study showed benefit of Impella 5.5 following IABP support failure. After the switch to Impella, these patients achieved functional recovery enabling participation in physical therapy, occupational therapy, or ambulation with improved cardiorenal syndrome or improved type 2 pulmonary hypertension, followed by a successful heart transplant, which supports the potential advantages of extensive Impella 5.5 use in this setting.

To date, studies for Impella protected cardiac surgery have been limited to small case series, with the majority reporting no to minimal mortality or morbidity (27–29). Collectively, these studies concluded that prophylactic use of the Impella 5.5 is safe and effective in patients with severe LV dysfunction. Once hemodynamics deteriorates in surgical cases with severe LV dysfunction, RV also fails with significant increase in preload and afterload, and eventually VA-ECMO is required for profound biventricular dysfunction. Unfortunately, the outcome for postcardiotomy cardiogenic shock (PCCS) requiring VA-ECMO is poor with a high rate of complications (30). In contrast, left ventricular unloading with Impella decreases wall tension, improves coronary perfusion favoring myocardial recovery, and could reduce pulmonary congestion and RV afterload with smaller bore access. In line with these data, the current study demonstrates that use of Impella 5.5 was protective and all surgical patients survived to discharge without any major device-related complications in our initial Impella 5.5 experience, despite sick population with 67% of cardiogenic shock status at stage C or D at the time of the cardiac surgery.

Interestingly, patients with CS that received an early pulmonary artery catheter (PAC) prior to MCS had improved short-term mortality and overall survival rates compared to patients without a PAC. This was also associated with lower incidence of short-term mortality, particularly in advanced CS (31, 32). Moreover, PAC-derived hemodynamic parameters such as CPO, pulmonary artery pulsatility index (PAPi), or CVP/PCWP ratio have been used to assess RV function, LV filling status, and guide treatment after Impella implantation. In Impella-supported cardiac surgery, 3–5 days are generally required to stabilize volume status and achieve organ perfusion at which point Impella support can start to be withdrawn. In our study, the mean support time of Impella 5.5 was 6.4 days for the patients that received Impella-supported cardiac surgery. Hemodynamic monitoring with PAC is an essential part of our practice to evaluate and manage both LV and RV dysfunction appropriately. A low threshold is set for longer MCS support to take advantage of axillary Impella, to reduce the requirement for vasopressors/inotropes, to avoid kidney dysfunction related to organ malperfusion, and to enable early extubation/early ambulation maintaining functional status without cardiac stress.

During critical illness, patients who are immobilized for more than a few days develop neuromuscular weakness despite receiving full supportive care. Thus, early mobilization (EM) of ICU patients is necessary to attenuate critical illness-associated muscle weakness (33) and is especially important to improve functional status for those awaiting heart transplant (34). At our institution, the mobilization protocol includes appropriate pain control with minimal sedation/narcotics; early extubation (in the operating room if possible); early tracheostomy if needed; physical therapy/occupational therapy consultation on post-operative day 0; active/passive range of motion even if intubated; out of bed to chair for all meals, ambulation twice or three times a day on post-operative day 1; cycle ergometer as needed; daily physical therapy/occupational therapy evaluation; and daily nutrition assessment.

Of the 6 cases of Impella access site/axillary wound re-exploration for hematoma, none had any active surgical bleeding at the time of re-exploration. We set a low threshold for wound exploration for hematoma/bleeding with consideration of risk of infection related to hematoma, especially for patients waiting for transplant. This strategy may have contributed to the relatively higher rate of bleeding related complication in this series. Other complications were minimal and included acute kidney injury or required renal replacement therapy (6 patients), transient high pf-Hb level (>20 mg/dL, 7 patients), device dislodgement (1 patient), and cerebrovascular accident (CVA, 2 patients). The CVA complications occurred during urgent Impella exchange cases as a result of significant hemolysis with groin Impella. Both had unclear neurologic status while on the ventilator throughout the procedure. There was left ventricular thrombus at the time of Impella CP insertion, which disappeared on follow-up TTE prior to Impella 5.5 device exchange. Thus, the CVA events likely happened prior to Impella 5.5 placement and were not related to Impella 5.5. Aortic valve injury, distal limb ischemia, or other vascular complications did not occur in this series.

**Figure 3:**
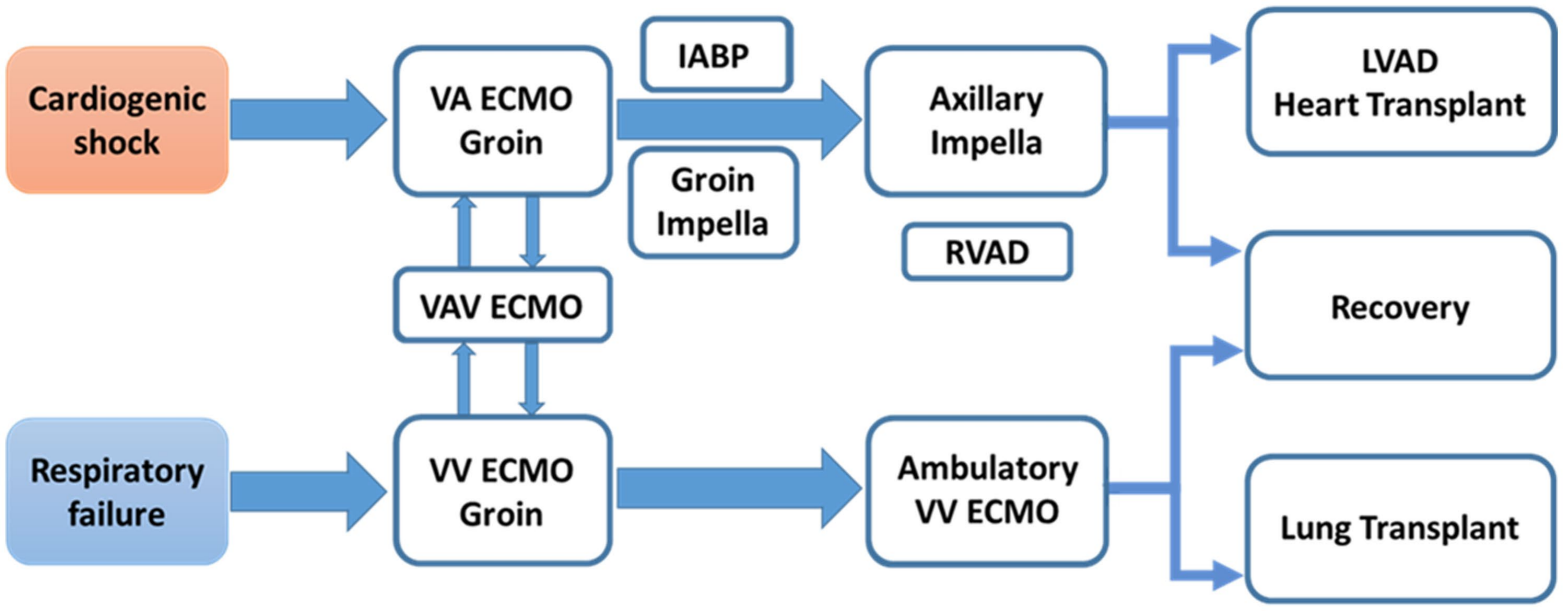
MCS de-escalation in patients with CS or heart failure. MCS is not just one time procedure. A team require comprehensive MCS experiences to provide optimal MCS strategy, tailored for patient’s status, and maximal survival benefit. IABP, intra-aortic balloon pump; LVAD, left ventricular assist device; MCS, mechanical circulatory support; RVAD, right ventricular assist device; VA-ECMO, veno-arterial extracorporeal membrane oxygenation; VAV ECMO, veno-arterio-venous extracorporeal membrane oxygenation; VV ECMO, veno-venous extracorporeal membrane oxygenation.

In the current MCS era, tailoring the MCS de-escalation approach according to patient condition is essential for best practices in patients with CS or heart failure (Figure 3). In this single center cohort, patients receiving Impella 5.5 support have meaningful outcomes in the management of profound CS and end-stage heart failure. Impella 5.5 is an important hub MCS device and can be used for up- or downgraded support and to bridge to the next treatment course. This includes LVAD and heart transplant, and the exchange from other temporary MCS inserted *via* the femoral artery (VA-ECMO, IABP or earlier Impella models) for longer duration support with less device-related complications or access site issues. In all indications, early MCS initiation strategy is crucial for maximal treatment effects and to avoid poor outcomes. Impella 5.5 utilization is considered at our institution if the patient needs more than 48-h MCS support at the time of evaluation.

## Limitations

Our study is not without limitations as this is a retrospective cohort study without randomization, with a small sample size that consists of a variety of patients with relatively stable CS and profound CS requiring VA-ECMO from a single center, and only reports short-term outcomes from temporary Impella 5.5 support. Additionally, our institution has more males than females that receive advanced surgical heart failure therapy and heart transplants (35). Thus, our institutional gender distribution also affects the gender disparity in this study. Furthermore, our cohort was not compared to patients treated with other MCS, such as IABP alone or other Impella devices (CP/5), or those treated with medical therapy only. Due to the nature of our retrospective study, PAC data and lactate level are not included here as there was a large amount of missing data for those parameters. Given that our cohort had a large number of patients on VA-ECMO and that both hemodynamic parameters and the dose of inotropes/vasopressors are affected by the support level of VA-ECMO, further assessments are deferred to studies with a more focused patient population in a larger cohort. As a result, this analysis is hypothesis generating for future studies.

## Conclusion

In this study, patients with surgically implanted axillary Impella 5.5 have encouraging short-term survival rates, specifically in patients with CS or decompensated heart failure, which have historically high early mortality rates. Axillary placement of Impella 5.5 was used in a multitude of clinical indications, such as bridging strategy to durable support, implant LVAD and heart transplant, or perioperative support for high-risk cardiac surgery all with excellent outcomes. The execution of prospective, multicenter, randomized, long-term outcome studies, are warranted to further delineate the optimal patient profile, timing, and management of Impella 5.5 support.

## Data availability statement

The raw data supporting the conclusions of this article will be made available by the authors, without undue reservation.

## Ethics statement

The studies involving human participants were reviewed and approved by HCA Healthcare Internal Review Board committee members, who determined informed consent was not required for this retrospective study.

## Author contributions

MF and MO: study conception and design. MF: data collection, analysis, and interpretation of results. MF, CK, MK, YM, MS, and MO: draft manuscript preparation. All authors reviewed the results and approved the final version of the manuscript.

## Conflict of interest

The authors declare that the research was conducted in the absence of any commercial or financial relationships that could be construed as a potential conflict of interest.

## Publisher’s note

All claims expressed in this article are solely those of the authors and do not necessarily represent those of their affiliated organizations, or those of the publisher, the editors and the reviewers. Any product that may be evaluated in this article, or claim that may be made by its manufacturer, is not guaranteed or endorsed by the publisher.
